# Socioeconomic burden of septic re-revision surgeries for periprosthetic joint infections in Europe – a comprehensive analysis of re-revision costs

**DOI:** 10.5194/jbji-11-523-2026

**Published:** 2026-08-24

**Authors:** Dominik Szymski, Susanne Baertl, Nike Walter, Markus Rupp, Katja Hierl, Volker Alt

**Affiliations:** 1 Department of Trauma Surgery, University Medical Centre Regensburg, Regensburg, Germany; 2 Department of Trauma, Hand and Reconstructive Surgery, University Hospital Giessen, Giessen, Germany

## Abstract

**Introduction:** Periprosthetic joint infection (PJI) is a major cause of failure after total hip arthroplasty (THA) and total knee arthroplasty (TKA). While primary PJI treatment costs are increasingly recognized, the economic burden of septic re-revision surgery remains poorly defined. This study estimated septic re-revisions and the reimbursement burden across Europe. **Methods:** A payor-perspective health-economic model was applied to 30 European countries, combining 2023 Eurostat arthroplasty data with published infection rates to estimate PJIs and septic re-revisions (including after aseptic revisions), categorized as DAIR (debridement, antibiotics, and implant retention), one-stage, or two-stage procedures. Reimbursement used 13-country survey data plus GDP-based extrapolation; sensitivity analyses varied key parameters by ± 20 %. **Results:** In 2023, 2.29 million primary arthroplasties were performed in Europe, resulting in an estimated 22 794 PJIs and 8629 septic re-revisions. Total reimbursement costs were estimated at EUR 123.6 million (EUR 74.1 million THA; EUR 49.5 million TKA), split into EUR 111.6 million after primary PJI treatment and EUR 12.0 million after initially aseptic revision. Two-stage exchanges accounted for 82.8 % of expenditures and were the main cost driver; Germany had the highest national costs. A Monte Carlo probabilistic sensitivity analysis yielded a 95 % credibility interval of EUR 102.0–156.2 million. **Conclusion:** Septic re-revision surgery after arthroplasty represents a substantial economic burden in Europe, largely driven by two-stage exchange procedures. Reducing infection and treatment failure rates may provide major clinical and financial benefits: a 25 % reduction in PJI incidence could save EUR 27.9 million annually (22.6 %), and shifting half of two-stage cases to one-stage a further EUR 22.6 million (18.3 %).

## 1 Introduction

Periprosthetic joint infection (PJI) remains one of the most devastating complications after total hip and knee arthroplasty. Despite continuous improvements in surgical techniques, perioperative management, and infection prevention strategies, PJIs occur in approximately 1 % of primary arthroplasties and are a leading cause of early failure (Springer et al., 2017). Beyond the profound clinical consequences for affected patients – characterized by pain, functional impairment, repeated surgeries, and prolonged antimicrobial therapy – PJIs impose a substantial economic burden on healthcare systems (Lawrence et al., 2024; Szymski et al., 2024).

While the costs of primary PJI treatment have been examined in several national studies (Garrido-Gómez et al., 2013; Kasch et al., 2016; Kasch et al., 2017; Romanò et al., 2010; Serrier et al., 2021; Sousa et al., 2018), far less attention has been paid to the socioeconomic impact of septic re-revision surgeries. Treatment failure requiring recurrent revision after an initial PJI intervention occurs in up to 30 % of hip arthroplasty cases and more than 20 % of knee arthroplasty cases, often necessitating further complex surgical procedures (Becker et al., 2025; Resl et al., 2024). These re-revisions are associated with longer hospital stays, higher complication rates, and markedly higher treatment expenses compared with primary revision procedures (Rodriguez-Merchan, 2024; Yu et al., 2020). However, comprehensive European-wide analyses quantifying this financial burden are lacking.

Existing literature has focused on single-country data or primary PJI costs; heterogeneous healthcare systems across Europe make extrapolation difficult (Alt et al., 2025). Moreover, the additional burden of septic failure after aseptic revision arthroplasty has rarely been incorporated into economic models, despite representing a clinically relevant proportion of cases.

Therefore, a robust European-level health-economic evaluation is needed. The aims of this study were (1) to estimate the number of septic re-revisions after PJI and after initially aseptic revisions in hip and knee arthroplasty across Europe; and (2) to calculate the corresponding reimbursement burden under different treatment options.

## 2 Materials and methods

This study is a socioeconomic analysis of periprosthetic joint infections and subsequent septic re-revisions of hip and knee arthroplasties in Europe. The analysis is based on data from the Statistical Office of the European Union (Eurostat), previously published epidemiological literature on infection and failure rates, and national reimbursement data for PJI treatment (Springer et al., 2017; Szymski et al., 2024; Becker et al., 2025; Resl et al., 2024; Alt et al., 2025; Lenguerrand et al., 2022; Leta et al., 2015). This analysis follows CHEERS 2022 guidance (checklist in the Supplement). “Septic re-revision” denotes any revision for infection, whether the preceding revision was itself septic (failed PJI treatment) or aseptic; these two populations are analysed separately throughout.

### 2.1 Health-economic modelling

The health-economic model was developed from the perspective of healthcare payors to assess the financial impact on healthcare systems across European countries. The analysis focused primarily on the reimbursement burden borne by public healthcare systems, which represent the predominant funding mechanism in Europe. Reimbursement from public payors was assumed to approximate basic payments from the private sector, as the data obtained from the Statistical Office of the European Union (Eurostat) do not differentiate between public and private healthcare provision. This assumption can be considered conservative, since private healthcare delivery is generally associated with higher costs and reimbursement levels.

**Figure 1:**
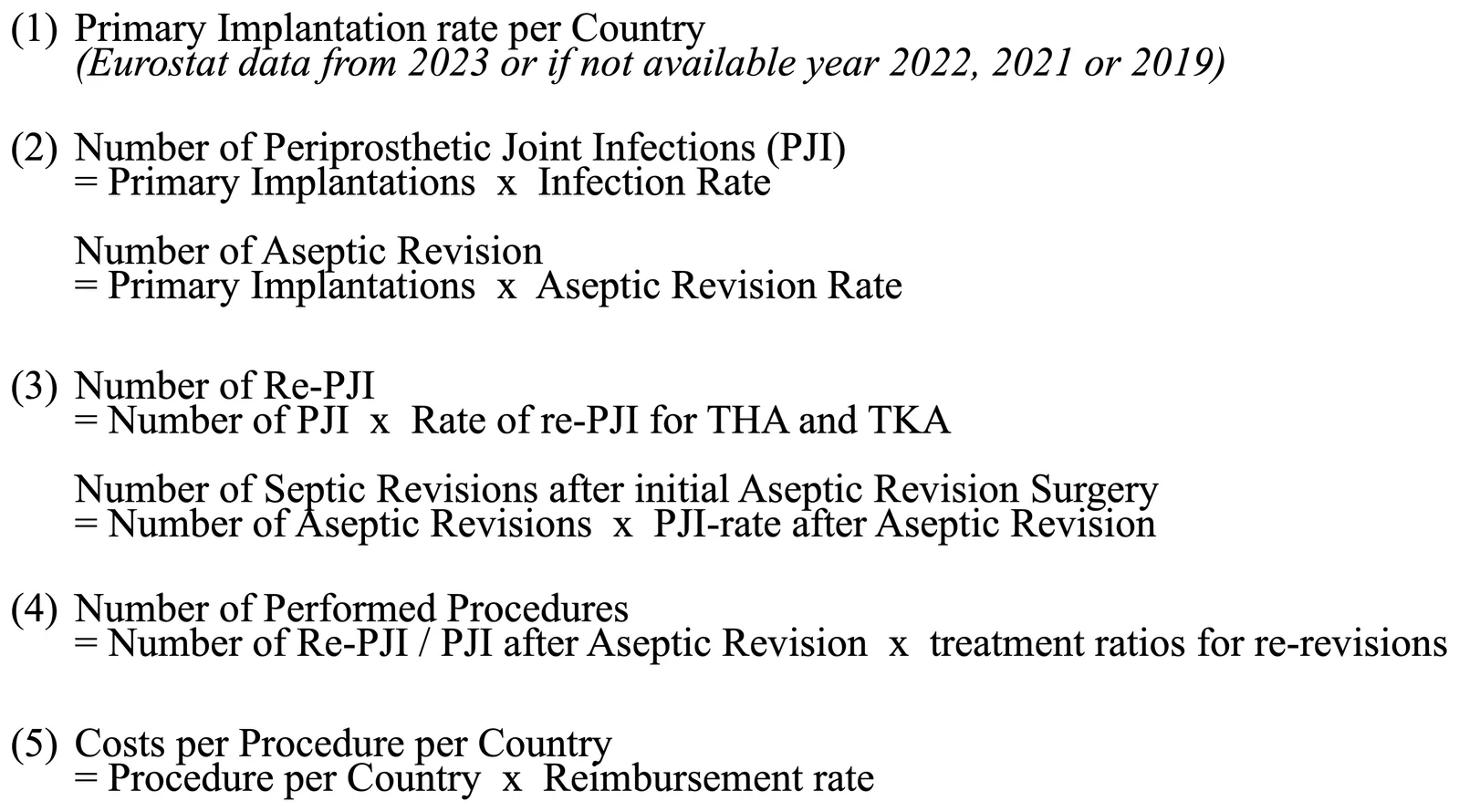
Study methods of estimation of re-revision procedures after periprosthetic joint infection and initial aseptic revision for total hip arthroplasty (THA) and total knee arthroplasty (TKA) in Europe in 2023.

The model followed a five-step calculation process (Fig. 1; Alt et al., 2025). First, Eurostat data from 2023 on the number of primary hip and knee arthroplasties were identified; if not available for 2023, data from previous years were utilized (Fig. 1, Eq. 1). Second, primary implantations were combined with published infection rates to estimate the total number of hip and knee periprosthetic joint infections (PJIs) and combined with published rates for aseptic revisions to identify the number of primary aseptic revisions across 30 European countries (Fig. 1, Eq. 2; Springer et al., 2017; Straub et al., 2024; Szymski et al., 2023). Third, the rate of septic revisions following PJI treatment and following initial aseptic revision surgery was estimated based on previously published rates (Fig. 1, Eq. 3; Becker et al., 2025; Resl et al., 2024; Lenguerrand et al., 2022; Leta et al., 2015). Fourth, the re-revision procedures performed were estimated based on published treatment ratios for septic re-revisions (Lenguerrand et al., 2022). Revision procedures were classified into three categories: (1) debridement, antibiotics, and implant retention (DAIR); (2) one-stage exchange; and (3) two-stage revision procedures (Fig. 1, Eq. 4). Fifth, these procedure volumes were multiplied by the corresponding reimbursement payments (Fig. 1, Eq. 5). Reimbursement data were obtained through a survey conducted in 13 countries (Austria, Croatia, France, Germany, Italy, Lithuania, the Netherlands, Norway, Portugal, Slovenia, Switzerland, Türkiye, and the UK) and extrapolated to the remaining countries using mean gross domestic product (GDP) values as reported in Alt et al. (2025). Eurostat data were the “Surgical procedures” dataset (hlth_co_proc3, extraction ID 19281055). The Lenguerrand et al. treatment strategy distribution (DAIR 3.8 %; one-stage 23.1 %; two-stage 70.4 %) was derived from a single English/Welsh registry and applied uniformly across countries; because practice mix varies across Europe, reported costs represent a uniform-practice projection rather than country-specific observed costs.

### 2.2 Calculation of reimbursement payments

Based on Alt et al. (2025), in the subsequent step, information on reimbursement payments received by hospitals from public healthcare funders for DAIR, one-stage, and two-stage PJI treatments was collected from 13 European countries through a survey coordinated by the EBJIS Country Delegates Group. For countries without direct data, a classification into higher- and lower-income groups was performed based on GDP per capita, sourced from Eurostat. GDP per capita ranged from 42 Purchasing Power Standards (PPS) in North Macedonia to 261 PPS in Luxembourg, with a European average of 101 PPS serving as the threshold for categorization (Table 1). Reimbursement amounts for each procedure type were multiplied by the corresponding number of interventions per country to estimate total payments per category (Fig. 1, Eq. 5). These were aggregated to national and European totals. Non-EUR costs were converted using 26 May 2023 exchange rates; public/private reimbursement equivalence was assumed for a conservative estimate. The GDP-tier extrapolation was validated by leave-one-out cross-validation (see Supplement).

A deterministic one-way sensitivity analysis was performed by varying key parameters by ± 20 %: (1) incidence of primary PJI, (2) septic failure rate after PJI treatment, and (3) proportion of two-stage exchange procedures. Changes in PJI incidence and failure rates affected only the number of septic re-revisions after PJI, while infections after aseptic revision remained constant. For treatment strategy distribution, the two-stage share was modified by ± 20 % relative to baseline, with the remaining proportion reallocated to one-stage procedures while keeping the total number of re-revisions unchanged. Total European reimbursement costs were recalculated for each scenario and compared with the base case.

To characterize compound parameter uncertainty, a probabilistic sensitivity analysis (10 000 Monte Carlo iterations; Beta-distributed rates, Dirichlet-distributed treatment mix, lognormal reimbursement values) was additionally performed; full distributional assumptions are detailed in the Supplement. The width of the resulting credibility interval is inherited directly from the ± 20 % relative uncertainty assumed for the Beta- and Dirichlet-calibrated epidemiological and treatment-mix parameters, except for reimbursement values in GDP-tier-extrapolated countries, where the lognormal multiplier instead uses the empirical leave-one-out cross-validation error (70.7 % THA, 80.8 % TKA) rather than the ± 20 % assumption.

**Table 1:** Costs for THA and TKA revision procedures from EBJIS survey data and income-class estimation.

Country	Source of costs	GDP per capita	Hip DAIR	Hip one-stage	Hip two-stage	Knee DAIR	Knee one-stage	Knee two-stage
		(PPS 2022)	(EUR)	(EUR)	(EUR)	(EUR)	(EUR)	(EUR)
Austria	EBJIS survey	114	10 500	11 000	27 000	10 500	11 000	27 000
Belgium	Estimation – higher-income class	120	13 885	15 209	29 456	12 814	16 944	30 737
Bulgaria	Estimation – lower-income class	59	6240	6986	11 207	4753	5325	9774
Croatia	EBJIS survey	76	7949	10 073	10 073	7949	11 267	11 267
Cyprus	Estimation – lower-income class	92	6240	6986	11 207	4753	5325	9774
Czechia	Estimation – lower-income class	91	6240	6986	11 207	4753	5325	9774
Denmark	Estimation – higher-income class	137	13 885	15 209	29 456	12 814	16 944	30 737
Estonia	Estimation – lower-income class	87	6240	6986	11 207	4753	5325	9774
Finland	Estimation – higher-income class	109	13 885	15 209	29 456	12 814	16 944	30 737
France	EBJIS survey	99	11 545	11 545	19 596	11 545	11 545	19 596
Germany	EBJIS survey	113	13 918	14 893	28 936	11 203	16 047	29 050
Hungary	Estimation – lower-income class	77	6240	6986	11 207	4753	5325	9774
Ireland	Estimation – higher-income class	233	13 885	15 209	29 456	12 814	16 944	30 737
Italy	EBJIS survey	96	8215	11 932	16 605	8215	11 932	16 605
Latvia	Estimation – lower-income class	68	6240	6986	11 207	4753	5325	9774
Liechtenstein	Estimation – higher-income class	280	13 885	15 209	29 456	12 814	16 944	30 737
Lithuania	EBJIS survey	82	3667	3667	3667	3580	3580	3580
Luxembourg	Estimation – higher-income class	261	13 885	15 209	29 456	12 814	16 944	30 737
Malta	Estimation – lower-income class	105	6240	6986	11 207	4753	5325	9774
Moldova	Estimation – lower-income class	40	6240	6986	11 207	4753	5325	9774
The Netherlands	EBJIS survey	125	18 113	17 985	36 097	14 720	18 050	34 013
North Macedonia	Estimation – lower-income class	42	6240	6986	11 207	4753	5325	9774
Norway	EBJIS survey	160	10 534	10 534	21 068	8715	8715	17 430
Poland	Estimation – lower-income class	80	6240	6986	11 207	4753	5325	9774
Portugal	EBJIS survey	88	5528	6986	11 415	4009	4009	13 793
Romania	Estimation – lower-income class	77	6240	6986	11 207	4753	5325	9774
Serbia	Estimation – lower-income class	44	6240	6986	11 207	4753	5325	9774
Slovakia	Estimation – lower-income class	68	6240	6986	11 207	4753	5325	9774
Slovenia	EBJIS survey	95	13 918	13 893	28 936	7249	7620	19 064
Spain	Estimation – lower-income class	85	6240	6986	11 207	4753	5325	9774
Sweden	Estimation – higher-income class	120	13 885	15 209	29 456	12 814	16 944	30 737
Switzerland	EBJIS survey	170	31 229	31 417	62 834	32 698	45 899	78 597
Türkiye	EBJIS survey	74	1331	4089	5266	978	3467	4483
United Kingdom	EBJIS survey	115	7028	12 365	23 608	4919	12 365	23 608

## 3 Results

### 3.1 Total number of hip and knee PJIs in Europe for primary arthroplasty performed in 2023

According to the data provided by Eurostat, the Statistical Office of the European Union for 2023, there were 1 003 002 primary TKA and 1 284 887 primary THA performed, with a combined total of 2 287 889 primary THA and TKA procedures in Europe. Most procedures were performed in Germany (THA: 292 143, TKA: 223 133, THA + TKA: 515 276), followed by France (THA: 184 264, TKA: 140 047, THA + TKA: 324 311), the UK (THA: 124 322, TKA: 98 651, THA + TKA: 222 973), and Italy (THA: 130 640, TKA: 101 274, THA + TKA: 231 914) (Tables 2 and 3).

Assuming infection rates for TKA and THA of 1.03 % and 0.97 %, respectively, as reported by Springer et al. (2017) in a review analysing PJI incidence rates from several international arthroplasty registries, the estimated total annual number of hip and knee PJIs is 22 794. This figure includes 12 463 hip PJI cases and 10 331 knee PJI cases, arising from primary THA and TKA performed in 2023 (Tables 2 and 3). These base-case rates already represent an unweighted average across six national/regional arthroplasty registries (AOANJRR, New Zealand, Swedish Hip and Knee Arthroplasty Registers, National Joint Registry, American Joint Replacement Registry) rather than a single-source estimate. A more recent registry-based estimate combining the Danish Knee Arthroplasty Registry with the Danish national infection surveillance system reported a cumulative 1-year PJI incidence of 0.8 %–1.1 % for TKA, closely consistent with the 1.03 % base-case value used here; this comparison is retained as a validation check rather than a base-case change, as fully registry-stratified rates by joint and fixation type (cemented vs cementless) were not available for all 30 countries at the time of this analysis. To quantify the impact of this range on the model rather than reporting it only as a qualitative validation check, we re-ran the TKA reimbursement calculation with the PJI incidence fixed at 0.8 % and at 1.1 %, holding all other parameters at their base-case values. TKA re-PJI reimbursement costs, EUR 41,299,232 at the 1.03 % base case, changed to EUR 32 077 073 ( - 22.3 %) at 0.8 % and EUR 44 105 976 ( + 6.8 %) at 1.1 %; propagated through the full model, the European grand total shifted from EUR 123.6 million to EUR 114.3 million ( - 7.5 %) and EUR 126.4 million ( + 2.3 %) at the low and high ends of the Danish range, respectively. As this sensitivity range lies within the ± 20 % deterministic bounds already reported above, we retain the Springer et al. (2017) base-case rate but present this arm explicitly rather than as a narrative aside.

Based on the reported septic re-revision rate after already performed surgical treatment of PJI of 30 % in THA and 21.7 % in TKA by Resl et al. (2024) and Becker et al. (2025), the number of septic re-revisions was 5981 (THA: 3739; TKA: 2242).

Simultaneously, to address septic re-revisions after an initial aseptic revision surgery in arthroplasties, a total of 23 882 aseptic revisions (THA: 12 849; TKA: 11 033) were identified, based on previous reported rates in the literature by Szymski et al. (2023) and Straub et al. (2024). Within the aseptic revision population, a total number of septic re-revisions of 648 (THA: 203; TKA: 445) was assumed based on Lenguerrand et al. (2022) and Leta et al. (2015) (Tables 2 and 3).

**Table 2:** Number of primary THA in Europe in 2023, with estimated primary PJI and aseptic revision cases, and number of re-revisions.

Country	Number of	Primary PJI	Primary aseptic	Re-PJI	Septic revision after	Total PJI
	primary THA	cases	revision cases	cases	initial aseptic revision	re-revisions
Austria	28 640	278	286	83	5	88
Belgium	33 956	329	340	99	5	104
Bulgaria	11 566	112	116	34	2	35
Croatia	7435	72	74	22	1	23
Cyprus	1454	14	15	4	0	4
Czechia	26 908	261	269	78	4	83
Denmark	18 005	175	180	52	3	55
Estonia	2835	28	28	8	0	9
Finland	16 570	161	166	48	3	51
France	184 264	1787	1843	536	29	565
Germany	292 143	2834	2921	850	46	896
Hungary	14 162	137	142	41	2	43
Iceland	793	8	8	2	0	2
Ireland	13 491	131	135	39	2	41
Italy	130 640	1267	1306	380	21	401
Liechtenstein	16	0	0	0	0	0
Lithuania	7199	70	72	21	1	22
Luxembourg	1333	13	13	4	0	4
Malta	438	4	4	1	0	1
Moldova	3630	35	36	11	1	11
The Netherlands	42 290	410	423	123	7	130
North Macedonia	1322	13	13	4	0	4
Norway	15 671	152	157	46	2	48
Poland	64 312	624	643	187	10	197
Portugal	12 116	118	121	35	2	37
Romania	17 605	171	176	51	3	54
Serbia	10 822	105	108	31	2	33
Slovakia	8621	84	86	25	1	26
Slovenia	5729	56	57	17	1	18
Spain	71 043	689	710	207	11	218
Sweden	29 560	287	296	86	5	91
Switzerland	30 991	301	310	90	5	95
Türkiye	55 005	534	550	160	9	169
UK	124 322	1206	1243	362	20	381
Total	1 284 887	12 463	12 849	3739	203	3942

**Table 3:** Number of primary TKA in Europe in 2023, with estimated primary PJI and aseptic revision cases, and number of re-revisions.

Country	Number of	Primary PJI	Primary aseptic	Re-PJI	Septic revision after	Total PJI
	primary TKA	cases	revision cases	cases	initial aseptic revision	re-revisions
Austria	21 417	221	236	48	9	57
Belgium	23 084	238	254	52	10	62
Bulgaria	3807	39	42	9	2	10
Croatia	3828	39	42	9	2	10
Cyprus	2066	21	23	5	1	6
Czechia	21 337	220	235	48	9	57
Denmark	15 622	161	172	35	7	42
Estonia	1693	17	19	4	1	5
Finland	14 714	152	162	33	7	39
France	140 047	1442	1541	313	62	375
Germany	223 133	2298	2454	499	99	598
Hungary	8554	88	94	19	4	23
Ireland	8385	86	92	19	4	22
Italy	101 274	1043	1114	226	45	271
Latvia	2160	22	24	5	1	6
Liechtenstein	13	0	0	0	0	0
Lithuania	3805	39	42	9	2	10
Luxembourg	1185	12	13	3	1	3
Malta	566	6	6	1	0	2
Moldova	2671	28	29	6	1	7
The Netherlands	22 490	232	247	50	10	60
North Macedonia	324	3	4	1	0	1
Norway	7239	75	80	16	3	19
Poland	38 717	399	426	87	17	104
Portugal	8779	90	97	20	4	24
Romania	4626	48	51	10	2	12
Serbia	3032	31	33	7	1	8
Slovakia	6559	68	72	15	3	18
Slovenia	3588	37	39	8	2	10
Spain	70 230	723	773	157	31	188
Sweden	18 149	187	200	41	8	49
Switzerland	28 297	291	311	63	13	76
Türkiye	92 960	957	1023	208	41	249
UK	98 651	1016	1085	220	44	264
Total	1 003 002	10 331	11 033	2242	445	2686

### 3.2 Estimation of DAIR, one-stage, and two-stage procedures per country

Based on the data of Lenguerrand et al. (2022), performed procedures in Europe were estimated for re-revisions, with 3.8 % being treated with a DAIR procedure, 23.1 % with a one-stage revision, and 70.4 % with a two-stage procedure.

By treatment strategy, THA re-revisions comprised 150 DAIR, 911 one-stage, and 2775 two-stage procedures; TKA comprised 102 DAIR, 621 one-stage, and 1891 two-stage procedures.

For THA, this comprised 142/864/2632 DAIR/one-stage/two-stage cases for re-PJI and 8/47/143 after aseptic revision.

For TKA, this comprised 85/518/1578 for re-PJI and 17/103/313 after aseptic revision.

### 3.3 Reimbursement payments for DAIR, one-stage, and two-stage PJI procedures per country

Reimbursement per procedure was taken from published country-specific billing data for 13 countries, as reported by Alt et al. (2025), stratified by surgical strategy and joint (Table 1).

### 3.4 Health-economic burden of septic re-revision after PJI and aseptic revision in THA and TKA in 2023 in Europe

For re-PJI treatment in hip arthroplasty performed in 2023, total reimbursement payments across all European countries were estimated at EUR 70 257 309, with the highest country-specific costs of EUR 20 692 501 in Germany. For septic revision after initial aseptic revision surgery, the total costs of EUR 3 814 947 in Europe in 2023 were identified.

Major drivers were two-stage revisions, with a total of EUR 58 224 587 in re-PJI of the hip and EUR 3 814 947 in septic revisions after aseptic revisions (Table 4).

In knee arthroplasty, total reimbursement payments for re-PJI treatments were EUR 41 299 231, with the highest country-specific costs in Germany (EUR 12 260 418) and France (EUR 5 290 410). Septic revisions after an initial aseptic revision of TKA accounted for EUR 8 190 765 in Europe in 2023. Simultaneously, two-stage revision was the major cost driver in TKA for re-PJI and septic revisions after initial aseptic exchange, with costs of EUR 34 167 010 and EUR 6 776 247, respectively (Table 5).

Overall, European healthcare systems are expected to face a total reimbursement burden of EUR 123 562 255 ( ≈ EUR 123.6 million) for septic re-revision surgery performed in 2023. Because the two source populations are epidemiologically distinct (see Methods), this total is reported as the sum of two separate estimates rather than a single aggregated figure: EUR 111 556 541 for re-revision following surgical treatment of primary PJI (THA EUR 70 257 309; TKA EUR 41 299 232) and EUR 12 005 714 for septic re-revision after an initially aseptic revision (THA EUR 3 814 948; TKA EUR 8 190 766). Of this total, the 13 directly surveyed countries (Austria, Croatia, France, Germany, Italy, Lithuania, the Netherlands, Norway, Portugal, Slovenia, Switzerland, Türkiye, and the UK) account for EUR 98 547 420 (79.8 %), with the GDP-tier extrapolation for the remaining 21–22 countries contributing the remaining EUR 25 014 835 (20.2 %); this subtotal is reported alongside the total, rather than in place of it, so that readers can judge the relative weight of directly observed versus extrapolated reimbursement data.

Varying PJI incidence or septic failure rates by ± 20 % changed overall costs to EUR 101.3–145.9 million ( ± 18.1 %); varying the two-stage share by ± 20 % changed costs to EUR 114.5–132.6 million ( ± 7.3 %). Fluctuations were larger for THA (EUR 68.6–79.6 million) than TKA (EUR 46.0–53.0 million), reflecting THA's greater share of baseline expenditure.

A country-level Monte Carlo PSA (10 000 iterations; Methods) yielded a median total of EUR 126.2 million (95 % credibility interval EUR 102.0–156.2 million; THA EUR 55.5–101.3 million, TKA EUR 38.2–66.6 million), consistent with the deterministic bounds above.

Leave-one-out cross-validation of the GDP-tier extrapolation showed a mean absolute percentage error of 70.7 % (THA) and 80.8 % (TKA), confirming GDP per capita is an imperfect proxy for individual-country reimbursement, although errors partially offset when aggregated to the European total (see Supplement).

Two counterfactual scenarios were modelled to illustrate the potential impact of prevention and treatment-pathway optimization. A 25 % reduction in PJI incidence (holding all other parameters at base-case values) reduced the projected total to EUR 95.7 million, a saving of EUR 27.9 million per year (22.6 %), avoiding an estimated 935 THA and 560 TKA re-revisions. Shifting 50 % of two-stage candidates to one-stage exchange (holding incidence constant) reduced the projected total to EUR 100.9 million, a saving of EUR 22.6 million (18.3 %). Combining both interventions reduced the projected total to EUR 78.2 million, a saving of EUR 45.4 million (36.8 %).

**Table 4:** Costs for treatment of re-revisions of THA in Europe (n/a – not available).

	Re-PJI				PJI after initial			
					aseptic revision			
Country	Costs	Costs one-stage	Costs two-stage	Total	Costs	Costs one-stage	Costs two-stage	Total
	DAIR	revision	revision	costs	DAIR	revision	revision	costs
Austria	33 252	211 766	1 584 126	1 829 146	1807	11 510	86 106	99 424
Belgium	52 135	347 147	2 049 025	2 448 307	2833	18 866	111 357	133 057
Bulgaria	7981	54 319	265 568	327 869	433	2953	14 438	17 825
Croatia	6536	50 353	153 457	210 347	353	2722	8296	11 372
Cyprus	1003	6826	33 373	41 202	54	371	1814	2240
Czechia	18 566	126 357	617 765	762 690	1007	6858	33 531	41 397
Denmark	27 642	184 060	1 086 412	1 298 115	1498	9977	58 893	70 369
Estonia	1956	13 313	65 090	80 360	106	726	3550	4383
Finland	25 442	169 410	999 939	1 194 791	1382	9204	54 331	64 918
France	235 240	1 430 015	7 397 330	9 062 586	12 770	77 633	401 589	491 993
Germany	449 625	2 924 722	17 318 154	20 692 501	24 413	158 803	940 322	1 123 539
Hungary	9771	66 503	325 135	401 410	531	3614	17 672	21 818
Iceland	1218	8115	47 902	57 237	68	456	2695	3221
Ireland	20 714	137 931	814 135	972 781	1123	7483	44 169	52 777
Italy	118 674	1 047 831	4 444 039	5 610 546	6443	56 889	241 279	304 613
Liechtenstein	26	175	1036	1238	n/a	n/a	n/a	n/a
Lithuania	2919	17 746	54 083	74 749	158	965	2942	4067
Luxembourg	2047	13 631	80 459	96 138	110	737	4354	5203
Malta	301	2049	10 019	12 370	16	112	552	681
Moldova	2503	17 041	83 315	102 860	135	919	4497	5552
The Netherlands	84 701	511 257	3 127 236	3 723 194	4597	27 752	169 754	202 104
North Macedonia	912	6213	30 375	37 501	49	338	1656	2045
Norway	18 253	110 960	676 333	805 547	992	6034	36 783	43 810
Poland	44 377	302 016	1 476 562	1 822 955	2409	16 395	80 159	98 964
Portugal	7406	56 901	283 355	347 663	401	3082	15 349	18 832
Romania	12 147	82 673	404 190	499 011	659	4486	21 933	27 078
Serbia	7466	50 817	248 447	306 731	405	2759	13 491	16 656
Slovakia	5949	40 489	197 953	244 392	322	2194	10 730	13 247
Slovenia	8816	53 498	339 583	401 898	481	2920	18 537	21 939
Spain	49 022	333 629	1 631 122	2 013 774	2660	18 106	88 522	109 289
Sweden	45 386	302 212	1 783 798	2 131 397	2464	16 407	96 841	115 712
Switzerland	107 016	654 465	3 989 124	4 750 607	5814	35 560	216 752	258 127
Türkiye	8095	151 186	593 384	752 666	439	8208	32 216	40 863
UK	96 617	1 033 349	6 012 745	7 142 712	5245	56 106	326 465	387 817
Total	1 513 731	10 518 990	58 224 587	70 257 309	82 194	571 162	3 161 591	3 814 947

**Table 5:** Costs for treatment of re-revisions of TKA in Europe.

	Re-PJI				PJI after initial			
					aseptic revision			
Country	Costs	Costs one-stage	Costs two-stage	Total	Costs	Costs one-stage	Costs two-stage	Total
	DAIR	revision	revision	costs	DAIR	revision	revision	costs
Austria	19 100	121 638	909 913	1 050 651	3787	24 114	180 386	208 287
Belgium	25 126	201 966	1 116 565	1 343 656	4981	40 041	221 365	266 388
Bulgaria	1537	10 468	58 556	70 561	305	2079	11 629	14 013
Croatia	2586	22 279	67 898	92 762	514	4425	13 484	18 422
Cyprus	834	5683	31 790	38 307	166	1132	6330	7628
Czechia	8613	58 662	328 150	395 426	1709	11 637	65 093	78 438
Denmark	17 004	136 679	755 629	909 311	3374	27 124	149 957	180 456
Estonia	683	4650	26 010	31 342	135	923	5161	6219
Finland	16 015	128 734	711 702	856 450	3175	25 520	141 085	169 780
France	137 325	834 791	4 318 294	5 290 410	27 235	165 561	856 430	1 049 226
Germany	212 312	1 848 684	10 199 422	12 260 418	42 107	366 645	2 022 828	2 431 581
Hungary	3453	23 519	131 563	158 535	685	4662	26 079	31 425
Ireland	9125	73 350	405 512	487 987	1811	14 560	80 497	96 868
Italy	70 663	623 914	2 646 130	3 340 707	14 013	123 730	524 761	662 504
Latvia	872	5941	33 235	40 048	173	1181	6606	7960
Liechtenstein	15	117	649	781	5	39	216	260
Lithuania	1156	7029	21 423	29 608	230	1398	4259	5887
Luxembourg	1290	10 372	57 343	69 006	258	2074	11 469	13 801
Malta	229	1562	8739	10 530	45	308	1720	2073
Moldova	1078	7344	41 079	49 501	213	1451	8119	9784
The Netherlands	28 119	209 603	1 203 723	1 441 445	5577	41 570	238 733	285 880
North Macedonia	130	886	4954	5970	25	172	963	1161
Norway	5358	32 573	198 540	236 472	1063	6462	39 389	46 914
Poland	15 630	106 451	595 473	717 554	3099	21 108	118 076	142 284
Portugal	2989	18 170	190 516	211 674	593	3602	37 773	41 968
Romania	1868	12 719	71 148	85 735	370	2522	14 106	16 998
Serbia	1225	8340	46 652	56 217	242	1648	9220	11 111
Slovakia	2648	18 033	100 874	121 555	526	3580	20 023	24 129
Slovenia	2209	14 117	107 637	123 963	438	2799	21 339	24 576
Spain	28 351	193 085	1 080 094	1 301 530	5623	38 292	214 202	258 117
Sweden	19 750	159 223	877 672	1 056 645	3920	31 601	174 193	209 714
Switzerland	78 590	670 619	3 499 767	4 248 976	15 581	132 957	693 867	842 406
Türkiye	7722	166 398	655 729	829 849	1532	33 004	130 060	164 596
UK	41 215	629 803	3 664 631	4 335 650	8174	124 912	726 827	859 914
Total	764 821	6 367 400	34 167 010	41 299 232	151 685	1 262 834	6 776 247	8 190 766

## 4 Discussion

The most important finding of the present study is that septic re-revision surgery after hip and knee arthroplasty represents a substantial and previously under-recognized economic burden for European healthcare systems. Based on current procedure volumes and published failure rates, more than 8000 septic re-revisions are expected annually across Europe, generating direct hospital reimbursement costs exceeding EUR 120 million. The majority of these expenditures were attributable to two-stage exchange procedures, which accounted for 82.8 % of total costs despite representing 70.4 % of all interventions.

These data demonstrate that treatment failure after an initial PJI intervention is not only a major clinical challenge but also a key driver of rising arthroplasty-related healthcare spending (Becker et al., 2025; Resl et al., 2024).

Previous health-economic analyses have largely focused on the costs of primary PJI management (Szymski et al., 2024; Alt et al., 2025). Several national studies have shown that two-stage revision is associated with the highest resource consumption due to prolonged hospital stay, repeated surgeries, and extensive antimicrobial therapy (Kasch et al., 2016; Serrier et al., 2021; Sousa et al., 2018; Assmann et al., 2014). The present analysis expands this knowledge by specifically addressing the re-revision setting at a European level. Patients requiring septic re-revision typically present with compromised bone stock, poor soft-tissue conditions, and multiple comorbidities, all of which increase surgical complexity and complication rates. Note that the high two-stage proportion driving costs reflects the input treatment strategy distribution (applied uniformly from a single registry), not an observation from the underlying European re-revision data (Lenguerrand et al., 2022).

A further relevant aspect of this study is the inclusion of septic failures after the initial aseptic revision arthroplasty. While most previous reports considered only reinfections after treatment of a primary PJI, registry data indicate that infection is also a frequent mode of failure following aseptic revision procedures (Lenguerrand et al., 2022; Leta et al., 2015). Our estimates suggest that this subgroup alone generates more than EUR 12 million in additional annual costs in Europe. Ignoring this component would therefore lead to a significant underestimation of the true socioeconomic burden of arthroplasty-related infections.

These findings have clinical and health-policy implications, underlining the importance of preventing not only primary PJI but also treatment failure. The optimization of DAIR patient selection is essential, as inadequate selection and delayed recognition of persistent infection are major drivers of early failure (Okafor et al., 2023); careful consideration of infection chronicity, implant stability, soft-tissue condition, and host comorbidities is needed to identify patients at higher risk who warrant alternative strategies (Sigmund et al., 2025). The cost differential between an appropriately selected DAIR, and a DAIR that fails and requires two-stage exchange is substantial (THA: EUR 10 654 vs EUR 32 774, TKA: EUR 8978 vs EUR 30 626), underscoring the fact that DAIR selection criteria carry direct economic implications. The centralization of care in specialized PJI centres with multidisciplinary teams may improve outcomes and reduce the need for costly re-revisions (Rupp et al., 2023; Walter et al., 2022; Winter et al., 2024). Second, the current reimbursement structures in many European countries may not adequately reflect the complexity of these procedures. Underfunding of re-revision surgery could threaten access to specialized treatment and disincentivize hospitals from accepting these high-risk patients (Alt et al., 2025).

Registry data confirm that septic revision and revision history are among the strongest predictors of re-revision, with risk concentrated in the first postoperative year (Becker et al., 2025; Resl et al., 2024), underscoring the value of close early postoperative surveillance.

Several limitations must be considered when interpreting the results. The model relied on uniform infection and failure rates derived from the literature, although true incidences vary between countries, hospitals, and patient populations. Reimbursement data were directly available for only part of Europe; for the remaining countries, GDP-based extrapolation was used, which cannot capture all national specificities of reimbursement systems and, in leave-one-out validation, produced a mean absolute percentage error of 71 %–81 % per country (Supplement). The model was not validated against an independent national cost dataset (e.g. German InEK DRG statistics), which we recommend for future collaborative work with national registries. Only direct hospital payments were considered; outpatient care, rehabilitation, antimicrobial therapy, productivity loss, and quality-of-life costs were excluded. From a societal perspective, the true burden is likely considerably higher than the EUR 123.6 million reported: applying the 2- to 4-fold underestimation factor reported for indirect costs in PJI modelling (Parisi et al., 2017), the societal-perspective burden may plausibly reach EUR 247–494 million annually (illustrative estimate). The study focused on DAIR, one-stage, and two-stage procedures; resection, arthrodesis, or fistula formation could not be included due to insufficient data.

Despite these limitations, the study provides the first comprehensive European estimate of the financial consequences of septic re-revision surgery after hip and knee arthroplasty. The results highlight the fact that failure after PJI treatment is not a rare exception but a frequent event (Becker et al., 2025; Resl et al., 2024), with major economic repercussions. Future research should aim to generate country-specific real-world cost data to evaluate the cost-effectiveness of preventive strategies and to identify modifiable risk factors for treatment failure. Reducing the incidence of septic re-revisions would not only improve patient outcomes but also offer substantial savings for European healthcare systems.

## 5 Conclusions

Septic re-revision surgery following hip and knee arthroplasty constitutes a major socioeconomic challenge in Europe, resulting in annual hospital reimbursement costs exceeding EUR 120 million. Two-stage exchange procedures are the predominant cost driver. In addition to failures after primary PJI treatment, infections occurring after initially aseptic revisions contribute substantially to this burden.

These findings emphasize the urgent need for effective prevention strategies, optimized treatment algorithms, and adequate reimbursement structures. Investment in specialized PJI care and research aimed at reducing re-revision rates has the potential to yield significant clinical and economic benefits for European healthcare systems.

## Supplement

10.5194/jbji-11-523-2026-supplement The supplement related to this article is available online at https://doi.org/10.5194/jbji-11-523-2026-supplement.

## Data Availability

The model draws on three categories of input data with different access statuses: (1) primary arthroplasty volumes are public Eurostat data (dataset hlth_co_proc3, custom extraction ID 19281055, https://ec.europa.eu/eurostat, last access: 20 April 2026), freely accessible without restriction; (2) epidemiological rates (infection, failure, and treatment strategy proportions) are taken from the published, peer-reviewed literature cited throughout the paper and are publicly available via the respective journals; (3) country-specific reimbursement values were collected through a survey coordinated by the EBJIS Country Delegates Group (see Alt et al., 2025). Reimbursement values were recorded and are retained only in euro as reported by country delegates, and the original local-currency figures underlying these conversions were not retained by the authors.
