# Supplementary material for: Socioeconomic burden of septic re-revision surgeries for periprosthetic joint infections in Europe – a comprehensive analysis of re-revision costs
Source: J Bone Jt Infect. 2026 Aug 24;11(4):523–33. doi: 10.5194/jbji-11-523-2026 (PMC13528776; doi:10.5194/jbji-11-523-2026)
Supplement: Supplementary file 1 [file jbji-11-523-2026-supplement.pdf]

Supplement of J. Bone Joint Infect., 11, 523–533, 2026
https://doi.org/10.5194/jbji-11-523-2026-supplement
© Author(s) 2026. CC BY 4.0 License.

Supplement of

Socioeconomic burden of septic re-revision surgeries for periprosthetic joint infections in Europe – a comprehensive analysis of re-revision costs

Dominik Szymski et al.

Correspondence to: Dominik Szymski (dominik.szymski@ukr.de)

The copyright of individual parts of the supplement might differ from the article licence.

S1. Probabilistic sensitivity analysis (PSA)

To characterize compound parameter uncertainty more comprehensively than the deterministic one-way analysis, a country-level probabilistic sensitivity analysis was performed. For each of the 34 European countries with 2023 primary arthroplasty volumes, the following distributions were assigned:

- **PJI incidence, septic failure rate after PJI treatment, aseptic-revision rate, and aseptic-to-septic conversion rate:** Beta distributions centered on their literature-derived point estimates (THA PJI 0.97%, TKA PJI 1.03%; THA septic failure rate 30.0%, TKA 21.7%; THA aseptic-revision rate 1.00%, TKA 1.10%; THA aseptic-to-septic conversion 1.58%, TKA 4.03%), with relative standard deviation calibrated so that the ±20% deterministic bound approximated the 95% interval (SD = 0.20/1.96 ≈ 10.2% relative).
- **Treatment-strategy distribution (DAIR / one-stage / two-stage):** a Dirichlet distribution centered on the Lenguerrand et al. (2022) proportions (3.8% / 23.1% / 70.4%), with concentration parameter S = 39.4, calibrated to reproduce the manuscript's own two-stage ±20% deterministic sensitivity bound.
- **Country-level reimbursement values:** lognormal multipliers (mean ≈ 1) applied to each country's base per-procedure reimbursement. For the 13 surveyed countries, a relative standard deviation of 15% was used, reflecting within-country billing variability. For the 21 extrapolated countries, the relative standard deviation was set equal to the leave-one-out mean absolute percentage error (MAPE) obtained for the corresponding income tier and procedure (see S2, Table S1), so that simulated uncertainty directly reflects the validated extrapolation error rather than an assumed value.

Ten thousand Monte Carlo iterations were drawn (numpy default_rng, seed 20260701). In each iteration, PJI, septic-failure, aseptic-revision, and aseptic-to-septic-conversion probabilities were sampled once per stratum (THA/TKA) and applied to all countries; treatment-strategy proportions were sampled once per iteration and applied uniformly; reimbursement multipliers were sampled independently for each country and procedure. Per-country per-procedure cost was multiplied by the corresponding simulated case count and summed to the country, stratum, and grand total. Results were summarized by median and 95% credibility interval (2.5th–97.5th percentile).

Table S1. PSA results by stratum and source population

Component	Median (€ million)	95% CrI low (€ million)	95% CrI high (€ million)
**Grand total**	126.2	102.0	156.2
**THA total**	75.3	55.5	101.3
**TKA total**	50.3	38.2	66.6
**THA – re-revision after primary PJI**	71.4	51.9	97.1
**THA – septic re-revision after aseptic revision**	3.9	2.8	5.3
**TKA – re-revision**	42.0	30.4	57.3

**after primary PJI**			
**TKA – septic re-revision after aseptic revision**	8.3	6.1	11.4

S2. Leave-one-out cross-validation of the GDP-tier extrapolation

To assess the validity of extrapolating reimbursement values to countries without survey data, each of the 13 surveyed countries was iteratively removed from its GDP income tier (higher-income: Austria, Germany, Netherlands, Switzerland, Norway, UK; lower-income: France, Italy, Croatia, Lithuania, Portugal, Slovenia, Türkiye), the remaining tier members were re-averaged to form a leave-one-out prediction, and this prediction was compared with the held-out country's actual reported reimbursement value for each of DAIR, one-stage, and two-stage procedures.

Table S2. Leave-one-out validation results – THA (MAPE = 70.7%, range 7.3–536.4%)

Country	Tier	Actual (DAIR/1S/2S, €)	Predicted (DAIR/1S/2S, €)	% error DAIR/1S/2S	
**Austria**	Higher	10,500 / 11,000 / 27,000	16,164 / 17,439 / 34,509	+53.9 / +58.5 / +27.8	
**Germany**	Higher	13,918 / 14,893 / 28,936	15,481 / 16,660 / 34,121	+11.2 / +11.9 / +17.9	
**Netherlands**	Higher	18,113 / 17,985 / 36,097	14,642 / 16,042 / 32,689	−19.2 / −10.8 / −9.4	
**Switzerland**	Higher	31,229 / 31,417 / 62,834	12,019 / 13,355 / 27,342	−61.5 / −57.5 / −56.5	
**Norway**	Higher	10,534 / 10,534 / 21,068	16,158 / 17,532 / 35,695	+53.4 / +66.4 / +69.4	
**UK**	Higher	7,028 / 12,365 / 23,608	16,859 / 17,166 / 35,187	+139.9 / +38.8 / +49.0	
**France**	Lower	11,545 / 11,545 / 19,596	6,768 / 8,440 / 12,660	−41.4 / −26.9 / −35.4	
**Italy**	Lower	8,215 / 11,932 / 16,605	7,323 / 8,376 / 13,159	−10.9 / −29.8 / −20.8	
**Croatia**	Lower	7,949 / 10,073 / 10,073	7,367 / 8,685 / 14,248	−7.3 / −13.8 / +41.4	
**Lithuania**	Lower	3,667 / 3,667 / 3,667	8,081 / 9,753 / 15,315	+120.4 / +166.0 / +317.6	
**Portugal**	Lower	5,528 / 6,986 / 11,415	7,771 / 9,200 / 14,024	+40.6 / +31.7 / +22.9	

**Slovenia**	Lower	13,918 / 13,893 / 28,936	6,372 / 8,049 / 11,104	−54.2 / −42.1 / −61.6	
**Türkiye**	Lower	1,331 / 4,089 / 5,266	8,470 / 9,683 / 15,049	+536.4 / +136.8 / +185.8	

Table S3. Leave-one-out validation results – TKA (MAPE = 80.8%, range 0.2–625.1%)

**Country**	**Tier**	**Actual (DAIR/1S/2S, €)**	**Predicted (DAIR/1S/2S, €)**	**% error DAIR/1S/2S**	
**Austria**	Higher	10,500 / 11,000 / 27,000	14,451 / 20,215 / 36,540	+37.6 / +83.8 / +35.3	
**Germany**	Higher	11,203 / 16,047 / 29,050	14,310 / 19,206 / 36,130	+27.7 / +19.7 / +24.4	
**Netherlands**	Higher	14,720 / 18,050 / 34,013	13,607 / 18,805 / 35,137	−7.6 / +4.2 / +3.3	
**Switzerland**	Higher	32,698 / 45,899 / 78,597	10,011 / 13,235 / 26,220	−69.4 / −71.2 / −66.6	
**Norway**	Higher	8,715 / 8,715 / 17,430	14,808 / 20,672 / 38,454	+69.9 / +137.2 / +120.6	
**UK**	Higher	4,919 / 12,365 / 23,608	15,567 / 19,942 / 37,218	+216.5 / +61.3 / +57.6	
**France**	Lower	11,545 / 11,545 / 19,596	5,330 / 6,979 / 11,465	−53.8 / −39.5 / −41.5	
**Italy**	Lower	8,215 / 11,932 / 16,605	5,885 / 6,915 / 11,964	−28.4 / −42.0 / −28.0	
**Croatia**	Lower	7,949 / 11,267 / 11,267	5,929 / 7,026 / 12,854	−25.4 / −37.6 / +14.1	
**Lithuania**	Lower	3,580 / 3,580 / 3,580	6,658 / 8,307 / 14,135	+86.0 / +132.0 / +294.8	
**Portugal**	Lower	4,009 / 4,009 / 13,793	6,586 / 8,235 / 12,432	+64.3 / +105.4 / −9.9	
**Slovenia**	Lower	7,249 / 7,620 / 19,064	6,046 / 7,633 / 11,554	−16.6 / +0.2 / −39.4	
**Türkiye**	Lower	978 / 3,467 / 4,483	7,091 / 8,326 / 13,984	+625.1 / +140.1 / +211.9	

The mean absolute percentage error (MAPE) per procedure, used to parameterize the lognormal reimbursement uncertainty for extrapolated countries in the PSA (S1), was: THA – DAIR 88.5%, one-stage 53.2%, two-stage 70.4% (overall 70.7%); TKA – DAIR 102.2%, one-stage 67.3%, two-stage 72.9% (overall 80.8%). These relatively large errors reflect substantial within-tier heterogeneity in national reimbursement systems (e.g. Switzerland's markedly higher costs, Türkiye's markedly lower costs, relative to tier peers) and confirm that GDP per capita is an imperfect proxy for reimbursement level at the level of an individual country. Errors partially offset when aggregated across the 21 extrapolated countries to obtain the European total, since over- and under-predictions occur in both directions within each tier.
